# Reading the National Disability and Rehabilitation Policy in the light of Foucault’s technologies of power

**DOI:** 10.4102/ajod.v2i1.41

**Published:** 2013-05-17

**Authors:** Lekholokoe P. Leshota

**Affiliations:** 1 National University of Lesotho, Lesotho

## Abstract

In the area of disability studies, models have been at the centre of debates, influencing social policies, practices and legal frameworks. The former Ministry of Health and Social Welfare in the Kingdom of Lesotho was not an exception. In its efforts to tackle issues of disability, it produced *The National Disability and Rehabilitation Policy: Mainstreaming persons with disabilities into society* in 2011. This policy document is rooted in the social model and seeks to address long-standing problems and challenges of people with disabilities in the Kingdom. Using ideas from Foucault, particularly the technologies and regimes of power, which work through language and practice, this article examined ways in which people with disabilities are constituted through state knowledge and government policies, and concluded that these constructions form the basis for alienation and marginalisation in society.

## Introduction

Issues surrounding disability and people with disabilities have gone through different phases of conceptualisation and re-conceptualisation by societies, from ancient to modern, in different ways and intensities. The 1970s saw a growing interest in the subject, reflected in the number of research articles and books across the spectrum of disciplines (Calderbank 2000; Tan 2004). The inclusion of people with disabilities into the mainstream of social life and academia changed the landscape of research on and ownership of issues of disability. This inclusion was further accompanied not only by diversification in research and advocacy on disability but also by campaigns and organisations spearheaded, run and controlled by people with disabilities (Gabel & Peters 2004; Shakespeare & Watson 1997). This has contributed to the dramatic change in perspectives on disability.

The above notwithstanding, disability has remained a complex phenomenon. This complexity has been documented well (Barton 1992; Powell 2003) and the debates surrounding its culturally variable and highly contested nature have been appreciated (Devlieger, Rush & Pfeiffer 2003). Throughout the years, the conceptualisation of disability has been held together by the ‘passion for sameness’ at the expense of ‘love for difference’ (Stiker 1999). According to Stiker (1999:ix), a ‘passion for sameness’ has occasioned the segregation and marginalisation of people with disabilities and their exclusion from mainstream society.

This marginalisation has been patent in how various institutions of state, particularly in developing countries, have handled the issues of disability and people with disabilities. The present article acknowledges that people with disabilities in Lesotho have for years, since the foundation of Disability People’s Organisations (DPOs), demanded a fair share in the opportunities that are afforded every citizen. But this demand has been met with a litany of broken promises which at the same time continued to construct people with disabilities as add-ons who can only be assisted when everybody else have been assisted. These constructions have influenced people’s perceptions about disability and people with disabilities and continue to do so. The policies that are formulated and their implementation are foregrounded in the perceptions that are informed by people’s constructions about disability and people with disabilities.

## Research methodology and framework

This article is concerned with how Lesotho’s former Ministry of Health and Social Welfare (MOHSW), through the National Disability and Rehabilitation Policy (NDRP) entitled Mainstreaming persons with disabilities into society (MOHSW 2011), constructs disability and people with disabilities through the language it employs. It is therefore a desktop study which comprises, for the most part, a review of existing published literature relating to issues of disability and people with disabilities. It examines the power valences inherent in the language used in the policy document, ministerial and departmental policies and other documents that bear on the issues under discussion. Using ideas from Foucault, particularly that of the technologies and regimes of power, which work through language and practice (Foucault 1991; Rose 1997), the article examines ways in which people with disabilities are constituted through state knowledge and government policies.

### Disability and the welfare system

Globally, economic, social and political factors have influenced the development of welfare systems with the aim of examining the welfare of those citizens who do not meet the requirements of a militarily, industrially and economically viable human resource within a competing capitalist economy within countries and nations (Drake 2001). Social concerns such as poverty, suffering and proliferation of vulnerable groups have also played their role in the development of welfare systems. Grönvik (2007:14) opines that the main task of the welfare state is to count the numbers in view of distributing support to some people, as well as providing justification for not giving it to others. It achieves that through delimiting categories of people eligible for certain grants and support through the process of assessment (Swartz & Schneider 2006). People with disabilities, worldwide, have always been regular clients of welfare systems. Through diagnosis, labelling and ascription, which entitle them to what Campbell (2003:167) calls an enumerative passport, they are rendered genuine people with disabilities through state apparatus. In that way they are classified as essentially disabled. While this may be seen by some as absolutely necessary to facilitate administration of disability through counting, it is seen by others as a re-invention of the medical model with a more sophisticated face (Anderberg 2006).

#### The Lesotho Department of Social Welfare

In Lesotho, the Department of Social Welfare was first established in 1976, as a way of responding to increasing levels of poverty and other social problems (Nyanguru 2003). It was first housed within the then Ministries of Internal Affairs, Justice and then Employment, before being transferred, in 1993, to the Ministry of Health and Social Welfare (MOHSW). According to Nyanguru (2003), its six moves in 17 years are indicative of the low status afforded the Department, which together with a long-standing lack of departmental policy has left its service provision fragmented, dispersed and lacking in focus. This consequently impacted negatively on the extent to which the Department was able to deliver services to its intended clients.

However, there are positive indications that point to a switch to improving the lot of people with disabilities. Firstly, the draft National Disability and Rehabilitation Policy of 2008 was made policy in 2011. Secondly, the establishment of the new Ministry of Social Development is a positive development and a realisation of an idea that was conceived in an effort to improve on the output of the Department of Social Welfare. The National Disability and Rehabilitation Policy 2011 (NDRP) read together with the Draft Disability and Rehabilitation Policy 2008 (DNDRP) constitute the foci of the analysis below.

#### The NDRP foregrounded in the social model

One positive development is that the Department of Social Welfare under the former Ministry of Health and Social Welfare spearheaded a formulation of a National Disability and Rehabilitation Policy (NDRP 2011). This document serves as major resource in the ensuing discussions. It is a very ambitious document which serves to give direction to the delivery of services, creation of opportunities and inclusion of people with disabilities in mainstream society. In keeping with the international trends in disability, the policy aligns itself with the in-vogue social model of disability, which situates the problem away from the individual and towards society. It is further informed by the constitution of Lesotho, various conventions, regional and international legal frameworks, as well as important national policies and legal structures such as the *Education Act* 1995, Section 3; *Local Government Act* 1997, Section 5 (1) and (2); *National Assembly (Amendment) Act* 2001; and *Children’s Protection and Welfare Bill* 2005, Clause 12.

The adoption of a social model marks an important theoretical and practical shift from the individualistic medical model (old paradigm) with its emphasis on diagnosis and treatment or elimination of a condition (Gathiram 2008). Instead it embraces a view that disability is a natural and normal part of human experience that in no way diminishes a person’s right to participate fully in all aspects of life (MOHSW 2008, 2011). It works towards the elimination of the environmental, institutional, attitudinal and economic barriers that prevent people with disabilities from participating meaningfully in society (MOHSW 2011). Situating the policy formulation within the framework of the social model will also curb the temptation, inherent in the location of the DPOs within the MOHSW, to view disability as an exclusive preserve of the medical and welfare professions.

The policy states clearly that disability is a human rights and developmental issue, a view that lends itself to sustainable and people-centred development (Gathiram 2008). To buttress mechanisms for achieving objectives of this developmental approach, the Community-Based Rehabilitation (CBR) strategy has been adopted, with the potential, if followed through well, to yield good results in the rehabilitation, equalisation of opportunities and social integration of people with disabilities (Gathiram 2008). Its community-based, participatory and action-oriented nature has made it better placed to enhance ownership, agency and accountability of programmes geared towards the integration of people with disabilities into society.

Mendis, Kachingwe and Khabele (2009:2) suggest, regarding Lesotho, that with cooperation and partnership it could move towards a coherent rights-based framework, with the MOHSW in a management role, the Lesotho National Federation of the Disabled (LNFOD) in advocacy and monitoring roles and local government structures in implementation roles. Such cooperation and clarification of roles would also help to stem the duplication of efforts that threatens to derail the social integration of people with disabilities (Mendis *et al*. 2009). It is the author’s opinion that even with this division of roles, people with disabilities have to participate at all levels, or at least be consulted at every stage. It would make absolutely no sense for management to conceive of ideas that are not informed by a lived experienced of people with disabilities only to be brought down to DPOs for approval, implementation and monitoring. This would undermine the spirit and principle of self-representation by people with disabilities that underlies the policy.

Despite the positive developments evident in the tone and orientation of the disability policy, anxieties remain. Gaps and rough edges of a theoretical and practical nature will always be there. These will be elaborated upon in the following sections.

### Conflicting perspectives: Social model and welfare agencies?

Though there is an obvious shift in perspective from the traditional medical model of disability and its paternalistic leanings in the NDRP (MOHSW 2011), anxieties associated with this not-so-distant, entrenched legacy remain. This legacy shows itself in very subtle ways in the document. The issue of the provision of social services in the form of welfare and grants is conspicuous in the policy document. The latter issue has a legacy that binds it to the paternalistic and patronising attitudes that were common of the medical model of disability. The biggest challenge, therefore, is how to balance the assumptive clash in perspectives between the social model and the welfarist tendency that remains within the new policy despite the felt need to change from welfarist to developmental orientation. In fact, the new policy (MOHSW 2011) has adopted a human rights and developmental approach within the framework of the social model of disability. This adoption marks an important break with not only the medical model, but also the long-standing tendency to forget people with disabilities through non-implementation of policies aimed at improving opportunities for them, only to patronise them through hand-outs and grants.

The focus of the social model is to point away from an individual with impairment to the society which disables him or her through limitations imposed by the same society. It targets removal of disabling barriers and advocates equality in opportunities and rights for people with disabilities (Albert 2004). In the concrete the social model advocates for removal of barriers, physical as well as attitudinal. It strives to enhance the educational opportunities of people with disabilities in order to maximise their ability and potential to compete equally with everybody else in the labour market.

On the other hand, welfare agencies were founded on the realisation that citizens do not have equal access to the country’s resources. Others, through no fault of their own, are vulnerable, poor and marginalised and therefore in need of some form of grant. If provision of social grants for individuals with disabilities, and who have been declared so through appropriate assessment procedures, is at the centre of machinations of the welfare state, the question is: how can this stance be reconciled with the social model stance which locates oppression in society and not in the individual? In other words, can the developmental approach, which aims at breaking economic dependency of people with disabilities (Gathiram 2008), be reconciled with a service-based approach, which creates the same dependency it intends to break from? The DNDRP (MOHSW 2008:16) recognises this theoretical quandary:

‘There is a need therefore for Government to provide social protection and disability grant to [*people with disabilities*] … Changing the way people regard disability from a purely health and welfare issue to a primarily human rights and development issue has significant implications for the principles, objectives and goals of existing welfare services. It implies that welfare services need to be designed to facilitate independence in society, rather than dependence on welfare services’.

Could this indicate that the policy is tending towards adoption of a model that combines social security with social and community development in line with international disability policy, where the focus has shifted from guaranteed income security towards economic integration (Mont 2004)? It appears that the policy balances theoretical considerations and pragmatic concerns. Within the framework of a social model, in which disability is seen more as a human right and developmental issue than an individual issue (Swartz & Schneider 2006), skills provision and creation of job opportunities are more important than disability grants. For disability activists the catchphrase is ‘human dignity and not separate services’.

Swartz and Schneider (2006:236) concur that the social model is founded on the assumption of a society that is as equal as possible for all. However, given gross poverty, inequality, inequitable distributions of resources, lack of skill development as well as high unemployment rates in Lesotho, application of a social model with a focus on creation of equal opportunities alone becomes a mammoth task. A stark reality to contend with is that people with disabilities invariably bear the brunt of these adverse consequences and would therefore, at some point, need social services in the form of grants.

### Assessment of needs: Whose needs?

The policy further foresees the need for the establishment of a multidisciplinary assessment team. The issue of assessment as regards people with disabilities has been regarded as given. The underlying assumption has always been that before a person can be said to qualify as authentically disabled some kind of a mechanism should be established to justify the selection of some and the rejection of others. The issue of an assessment of needs therefore is in order here. The true question is, however: whose needs?

As noted above, disability is a fluid concept. Its definition is dependent on who is attempting it and for what purpose. The Department of Social Welfare in Lesotho was founded with the purpose of attending to poverty and other social problems. That people with disabilities’ concerns are taken care of within this department suggest that disability is an issue that is in one way or the other associated with poverty or viewed as a social problem. Lesotho is rated among the poorest economies with high rates of unemployment and poverty, as well as differential access to resources (May *et al*. 2002). Although these needs are of a general nature, affecting the whole society, people with disabilities feel most the effects of poverty and marginal opportunities in the labour market.

Under these circumstances, their reasonable option is to wait for disability grants, but this is not as simple as identifying oneself as such and then receiving it. Rather, it involves a normal welfare process of diagnosis, normally referred to as ‘assessment’, which seeks to answer the question whether an individual qualifies to be categorised as disabled, and therefore deserving of a welfare benefit or disability grant (Swartz & Schneider 2006). Looked at very closely, the diagnostic assessment goes beyond serving only as a mechanism that helps administrators to distinguish ability from disability.

However, the assessment cannot be made without an assessment tool, otherwise such an assessment would depend on the whims of the person in office. Developing such a tool raises questions: Would the development of such a tool depend on the state of being of a person with disability or on the complex and changing environment (Swartz & Schneider 2006)? Who would have the last word on the development of such a tool and the criteria adopted in administratively identifying a person as disabled and therefore deserving of a disability grant? Whose needs are met by the development of such an assessment tool: the welfare authorities or people with disabilities?

The assessment tools are developed to ascertain the correctness of the decisions made about the welfare systems’ classification of ability and disability, so their purpose is to describe and classify. Assessment is also about constructing that which is described and classified, but classification also leads to apprehensions about who qualifies and who does not. Perhaps even more sensitive is the issue of who has the final say on who qualifies for a grant and who does not, on the basis of which norm is applied (Soudien & Baxen 2006). This sensitivity has to be understood in the light of the sentiment expressed by many people with disabilities and DPOs that projects are often written in their name but they are the last to enjoy the benefits. This sentiment, whether real or unreal, is an issue of power relations and justice, and calls for the re-examination of the kind of ethics that drive the interaction. It calls for a review of power valences that create the hierarchy between ‘us’ and ‘them’, with the ‘us’ responsible for the setting of norms and standards, and for the administration of disability grants. The ‘them’, meanwhile, can only be thankful or else they are dubbed ‘ungrateful’.

The tendency is nearly always to assume that the needs served are clearly those of people with disabilities, but this is not the case. There are two kinds of need here: those of the welfare authorities and those of people with disabilities. The welfare authority is interested in the proper administration of a welfare benefit, which can only be ascertained through an assessment procedure. A person with disability would like to be acknowledged as such and given his or her due. These needs do not have to clash, but they often do, and the people with disabilities usually benefit the least, if at all.

Through this procedure a person is labelled ‘administratively disabled’, which becomes a need that can be met by a welfare authority. Thomas and Loxley (2001:52) regard this case as one in which a welfare authority, ‘with a stroke of a wand’, is changed from assessor and labeller to benefactor and helper. Not only is there a change of roles but also a play of power valences, the effects of which are ‘hierarchizing, and forever, pushing x above y’ (Thomas & Loxley 2001:84). What Foucault (1991:308) terms a ‘disciplinary regime’ permeates ‘almost seamlessly and unquestionably the day to day workings of institutional life of people with disabilities’.

The NDRP does not yet have an answer to many of the above questions, but anticipates guidelines that would provide for the assessment of those who do and do not qualify for a social security grant.

### Rehabilitation of society or people with disabilities?

Community-based rehabilitation is adopted as a key strategy in achieving the objectives of the NDRP. Though a tested strategy, especially within the health sector, its relevance and appropriateness within the context of a socially oriented policy on disability still needs to be run through. The adoption of the language of rehabilitation within the policy is quite problematic and needs to be teased out. The questions that guide our reflection in this section are: what does rehabilitation mean? Who or what needs rehabilitation? Who does the rehabilitation and who stands to benefit from such an exercise? Are rehabilitation practices not a reconstitution of old discourses to resecure another centre from which to advance coercive practices in the government of disability?

### Rehabilitation language

**FIGURE 1:**
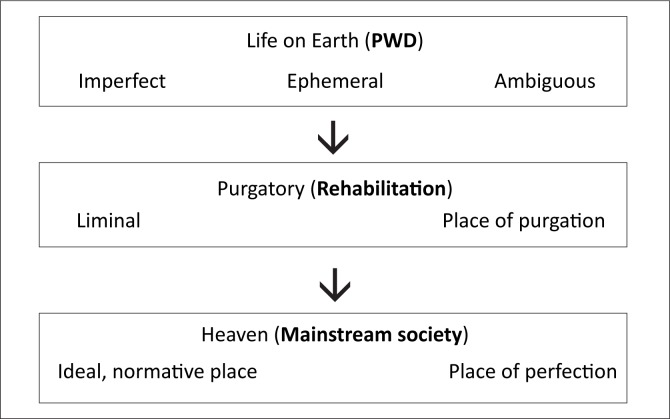
Heavenward stages.

The NDRP is replete with references to rehabilitation as an important *modus operandi* in addressing the plight of people with disabilities. The term ‘rehabilitation’, lexically, implies a return to a point or to a prior situation. Stiker (1999:122) suggests that this is the situation that existed for the able, but one postulated for the others. The whole understanding is premised on the centre, on the norm which has to be re-inhabited through the process of rehabilitation. This way of thinking can be likened to a traditional Catholic image of stages toward heaven, as represented in Figure 1.

The above diagram has three distinct stages. The first is life on earth, distinguished by its ephemeral nature, ambiguity and imperfection. In the middle is the stage of purgation (purgatory), a liminal stage where all dirt is eliminated before final incorporation or integration into the third stage, namely heaven. The latter represent the ideal, the norm and perfection in its purest sense. Following upon this analogy, people with disabilities represent an imperfect humanity, in its physical and moral sense, which has to go through some form of purgation (rehabilitation) before they can be included into mainstream society. According to Stiker (1999:136), this mainstream society sees itself as having the duty, mission and task of voiding disparities into its norm. Disability, according to this analogy, is sustained by the desire to flee from itself towards the mainstream. Until such a desire is fulfilled, disability cannot rest. If this is the understanding, as implied in the policy, then disability will forever remain the ‘different’ and the ‘alterity’ that must disappear (Stiker 1999:xii).

The language of rehabilitation is associated with the medical model of disability, stemming from the hospital (Stiker 1999). The adoption of the rehabilitation language within the NDRP, consciously situated within the social model, the new paradigm, is evidence of this medical shadow and the dominance of the medical model. Despite the intent to shift perspectives and nuances, the link between rehabilitation services and medicine is so glaring that any effort to divorce them becomes a futile exercise. The rehabilitation model as a substitute appellation for the medical model betrays this tendency. The trouble in shifting from one paradigm to the other is evident in this policy, and could create conceptualisation problems that are often part of working with and within models and paradigms. It begs the question as to whether one is working within the ‘new paradigm’ but with nostalgia for the ‘old paradigm’.

The policy defines rehabilitation as a means to *help* people with disabilities to fully participate as members of society (MOHSW 2008, 2011). One cannot fail to see the common power implications of helper (powerful) and helpee (vulnerable), doctor (powerful) and patient (vulnerable), which have been a subject of intense debate in the field of modern therapy (Van Wyk 2007).

Reading the definition of rehabilitation together with the objectives of rehabilitation as a priority policy area (MOHSW 2011), it becomes clear that the focus of rehabilitation is not society but people with disabilities. The objectives of rehabilitation are stated as promoting availability of the necessary skills and services to all people with disabilities, and enabling them to achieve and maintain their optimum physical, sensory and functional level. Nowhere under the objectives of rehabilitation is mention made of society as the object of the rehabilitation services. It is indisputable, therefore, that rehabilitation services are directed to people with disabilities, and that their accessibility and availability are made possible by the presence of rehabilitation professionals at all levels. McNamee (1996:145) uses the term ‘identity adjustment’ – which is what the medical model seeks to achieve – to refer to the process of rehabilitation.

This use of words evokes the relations of power between the rehabilitation service provider, who is skilled and equipped, and a person with disability, who is portrayed as lacking in something that must be filled by professional medical personnel (Stiker 1999). This does not seem to square up well with the social model of disability.

One of the objectives of rehabilitation as spelled out by the policy is to ‘enable [people with disabilities] to achieve and maintain their optimum physical, sensory, and social functional level’ (MOHSW 2011:6). The word ‘achieve’, used together with ‘optimum’, has a sense of ‘not yet there’. What would be the ‘not yet there’ compared to the present condition? Is it not suggestive of the undesirable state of disability compared to the desirable state of optimum physical, sensory and social functional level? On what basis does one measure that optimum and functional level, and who determines the achievement of that functional level? It evokes memories of the ideal, the normal into which the promise to restore an individual with disabilities comes alive. Is the promise to restore an individual to the ideal not a reassertion of the binaries of abnormal and normal? If answering in the positive, as I think I should, the binary logic harbours workings of power. It is founded on the moral and political hierarchy of the normal over the abnormal. This hierarchy, as Danforth and Rhodes (1997:359) assert, can be seen in the way the abundant social value accorded the first term is negatively mirrored in the corresponding devaluation of the second term.

By embracing this form of a rehabilitation discourse one is not far from the discourses of the late eighteenth and nineteenth centuries, in which restoring disabled people to a level of acceptable functionality was achieved through educational facilities and medical correction and technology (Stiker 1999). The definition of disability in the NDRP only helps to entrench this nostalgia. It further underlines power valences between people with disabilities and rehabilitation professionals through the language used (Stiker 1999).

Rehabilitation represents the medical gaze, as an eye of surveillance with immense power and an exclusive claim to knowledge, watching over and controlling people with disabilities (Foucault 1978). In the view of Oliver (1990), that is not appropriate because disability is not a medical condition but a social state, but I argue that it may not be necessary to pit one against the other in an either/or dichotomy. Read within a postmodern context, disability surpasses the social medical dichotomy and represents a complex and contingent variable that ‘describe[*s*] different aspects of a single experience’ (Shakespeare & Watson 2002:24). Care has to be taken therefore that the rehabilitation strategy embraced by the NDRP does not become a way of carting off the oppressive conditions of the medical model of disability through the front door only to bring them back through the back door.

However, this is not to suggest that people with disabilities do not have medical needs, but rather that the situation of a rehabilitative language, which insinuates the medical model, within the social model of disability is problematic. At issue here is whether there is a role for rehabilitation language and practice within the social model of disability. If, according to the social model, society has to change and not individuals, why should the language and practice of rehabilitation that target people with disabilities be dominant in a policy that adopts the social model as its guidepost? Perhaps, as Derrida (1976) would have suggested, we should put the word ‘rehabilitation’ under erasure to underline both its necessity and its inadequacy.

The Ministry of Health and Social Welfare, through its National Disability and Rehabilitation Policy 2011, straddles two paradigms, having adopted the social model with its one foot in the deficit and medical model. In the process, people with disabilities are constructed as ambiguous. Within the context of this research there is no intention to pit one model against the other, as both have their usefulness. The above discussion on the discourses within the Ministry of Health and Social Welfare in Lesotho reflects what in liberation theology is termed ‘social analysis’ (Lartey 2003:127). The aim is to explore ways in which power relations within different public institutions in Lesotho hold sway over the human person, particularly one with disability.

## Ethical considerations

As this article is a desk top critical reading of a policy document, the author did not have to do interviews which would have required ethical clearance. The views that are expressed are, therefore, the author’s own unless otherwise stated.

## Conclusion

Contesting notions and models of disability, informed by different theoretical underpinnings, have been at the centre of debates surrounding issues of disability. They have become a powerful force influencing social policies, practices and legal frameworks (Dewsbury *et al*. 2004). The above discussion has examined the extent to which government machinery, particularly in the Ministry of Health and Social Welfare, is influenced, in a disabling or enabling way, by these models. The constitution of Lesotho identifies the needs of people with disability as rehabilitation and resettlement (Lesotho Government 1993). It therefore identifies and constructs people with disabilities as ‘abnormal’ and in need of regularisation before they can be accepted into the fold of ‘the normal’. The MOHSW, through its NDRP, embedded in the social model, puts people with disabilities at the mercy of professionals and medical experts. The nature of the language adopted and used without critique, specifically in the NDRP, has further constructed people with disabilities as institutional subjects whose lives depend on the policies, laws and protocols of the powerful.

Through the use of Foucault’s ideas of governmentality and power, this paper has explored how policies and laws connive to create social meanings and power relations through language and models. Foucault’s ideas have served to unwind the structural composition of the Ministry of Health and Social Welfare as a public institution in terms of power relations. Power and knowledge combined in how health and welfare contributed to the construction of ambiguous but objectified disabled identities. Foucault’s ideas have also revealed that disability is sustained by social practices which serve the interest of dominant groups in society (Burr 2003), through constructing people with disabilities into ‘particular and shifting forms of objectification’ (Jolly 2003:517).
